# Development and evaluation of a Japanese prediction model for low anterior resection syndrome after rectal cancer surgery

**DOI:** 10.1186/s12876-022-02295-w

**Published:** 2022-05-13

**Authors:** Masakatsu Paku, Norikatsu Miyoshi, Shiki Fujino, Tsuyoshi Hata, Takayuki Ogino, Hidekazu Takahashi, Mamoru Uemura, Tsunekazu Mizushima, Hirofumi Yamamoto, Yuichiro Doki, Hidetoshi Eguchi

**Affiliations:** Department of Gastroenterological Surgery, Graduated School of Medicine, Osaka University, 2-2 E2, Yamadaoka, Suita, Osaka 565-0871 Japan

**Keywords:** Rectal cancer, Laparoscopy, LARS, ISR, Nomogram

## Abstract

**Background:**

Low anterior resection syndrome (LARS) is the most common complication after rectal cancer resection. We aimed to identify LARS' predictive factors and construct and evaluate a predictive model for LARS.

**Methods:**

This retrospective study included patients with rectal cancer more than 1 year after laparoscopic or robotic-assisted surgery. We administered a questionnaire to evaluate the degree of LARS. In addition, we examined clinical characteristics with univariate and multivariate analysis to identify predictive factors for major LARS. Finally, we divided the obtained data into a learning set and a validation set. We constructed a predictive model for major LARS using the learning set and assessed the predictive accuracy of the validation set.

**Results:**

We reviewed 160 patients with rectal cancer and divided them into a learning set (n = 115) and a validation set (n = 45). Univariate and multivariate analyses in the learning set showed that male (odds ratio [OR]: 2.88, 95% confidence interval [95%CI] 1.11–8.09, *p* = 0.03), age < 75 years (OR: 5.87, 95%CI 1.14–47.25, *p* = 0.03) and tumors located < 8.5 cm from the AV (OR: 7.20, 95%CI 2.86–19.49, *p* < 0.01) were significantly related to major LARS. A prediction model based on the patients in the learning set was well-calibrated.

**Conclusions:**

We found that sex, age, and tumor location were independent predictors of major LARS in Japanese patients that underwent rectal cancer surgery. Our predictive model for major LARS could aid medical staff in educating and treating patients with rectal cancer before and after surgery.

**Supplementary Information:**

The online version contains supplementary material available at 10.1186/s12876-022-02295-w.

## Background

Colorectal cancer is one of the most prevalent types of cancer worldwide [1]. Survival from colorectal cancer has increased over the past 30 years due to advances in surgery, chemotherapy, and other medicines [2]. The standard treatment for resectable colorectal cancer is radical resection. However, in recent years, total mesorectal excision and intersphincteric resection (ISR) have become widespread in treating rectal cancer [3, 4]. These techniques have reduced the local recurrence rate, but they have negatively impacted anorectal function [5, 6].

Surgery for rectal cancer, particularly low rectal cancer, frequently causes defecation disorders, with a reported incidence of 37–71% [7–9]. Defecation disorders after rectal cancer surgery are termed low anterior resection syndrome (LARS). The LARS score is an index used to assess the severity of LARS [10], and the LARS score has been correlated to the quality of life [11, 12].

Recently, a European group created a nomogram of a predictive model of LARS occurrence after rectal cancer surgery [13]. The nomogram could be an effective aid for preoperative education and counseling. However, they created the nomogram based on European patients with rectal cancer, and it is not known if the European nomogram is helpful for Japanese patients with the same condition.

The present study aimed to clarify the risk factors for developing LARS after rectal cancer surgery in Japan and establish a model for predicting LARS occurrence in Japanese patients.

## Methods

**Fig. 1:**
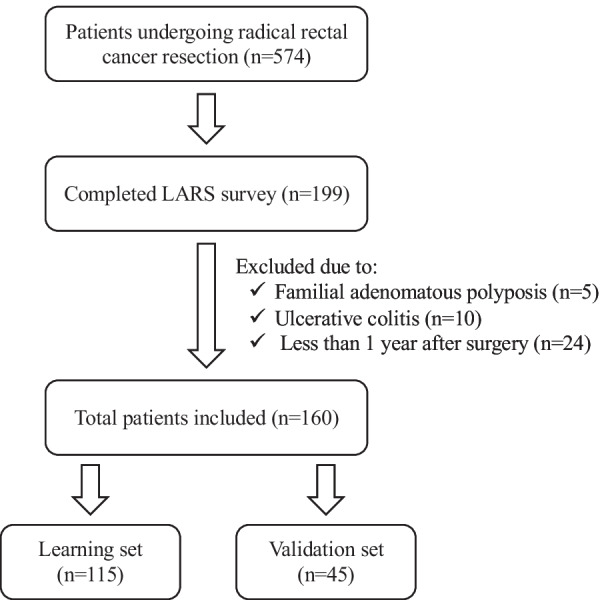
Patients selection process for this study

We retrospectively reviewed 574 patients who underwent radical rectal cancer resection with lymph node dissection between January 2010 and July 2019 at Osaka University Hospital. Among the patients, 199 were followed up in our outpatient clinic and completed our LARS survey questionnaire between April 2017 and July 2020. We excluded patients with familial adenomatous polyposis (5 cases) or ulcerative colitis (10 cases). We also excluded patients less than 1 year after surgery (24 cases). We finally included 160 patients in this retrospective study. We divided the obtained data into a learning set (n = 115, April 2017 to May 2019) and a validation set (n = 45, June 2019 to July 2020) due to the timing of the questionnaire (Fig. 1).

### Patient and tumor characteristics

We collected data on patient sex, age at surgery, body mass index (BMI), and the presence or absence of preoperative treatment. We also collected data on tumor characteristics, including the distance from the anal verge (AV) to the anal margin of the tumor and the pathological stage according to the TNM classification system (UICC 8th edition [14]). Furthermore, we collected data on the surgical procedure, surgery duration, and blood loss. Among patients with lower rectal cancer, we performed ISR if there was no invasion of the external anal sphincter or anal levator muscle.

### LARS survey

We scored the completed questionnaires and classified the severity of LARS according to a scale described previously [10]. Scores of 0–20 indicated no LARS, 21 to 29 indicated minor LARS, and 30 to 38 showed major LARS. We performed a multiple logistic regression analysis to identify the correlation between major LARS and the preoperative clinical factors.

### Statistical analysis

All categorical data are presented as the number of cases and percentages, while continuous data are shown as the median and interquartile range (IQR). We performed logistic regression analysis to assess the correlation between the incidence of major LARS and the distance from the AV to the anal margin of the tumor and to set the cut-off value. We also performed univariate analysis using the Mann–Whitney U test and multivariate analysis using logistic regression analysis to assess the correlation between major LARS and clinical factors.

The data were processed and analyzed with JMP Pro 14.0 (SAS Institute Inc., Cary NC). A two-tailed *p* value < 0.05 was considered statistically significant. To evaluate absolute differences, we calculate odds ratios (ORs) and exact 95% confidence intervals (95% CIs). We used only elements with statistically significant multivariate analysis differences to create a nomogram for major LARS with R statistical software (version 3.5.0).

We performed this study following the Declaration of Helsinki (1975, revised in 2008). The Ethics Committee at Osaka University Hospital approved the study protocol. All study participants provided written comprehensive informed consent. For observational studies using only anonymized medical data at the initial consultation, we provided opt-out statements for data collection and use.

## Results

### Clinical characteristics of the study population

Table 1 shows the patients, tumor characteristics, and surgical details of 160 patients with rectal cancer post-surgery. Among the 115 patients in the learning set, the cohort included 73 males (63.5%) and 42 females. The median age was 62 years (IQR 55–67), and 13 patients (11.3%) were older (age ≥ 75). The median BMI was 22.5 kg/m^2^ (IQR 20.4–24.5), and 23 patients (20.0%) were obese (BMI ≥ 25). A total of 21 patients (18.3%) received neoadjuvant therapy, including chemotherapy alone. The median tumor distance from the AV was 10 cm (IQR 6–15). About half the patients had a tumor stage of T3 or T4 (T3, n = 44, 38.3%; T4, n = 11, 9.6%). About half the patients had lymph node involvement (N1, n = 30, 26.1%; N2, n = 11, 9.6%), and 5 patients had metastases (4.3%). We performed laparoscopic surgery in more than 70% of patients (n = 91; 79.1%); robot-assisted surgery in the remaining 24 patients (20.9%). More than half the patients underwent low anterior resection (n = 60; 52.2%), and ISR was performed in 13 patients (11.3%). In 48 patients (41.7%), we constructed a diverting ileostomy and performed lateral lymph node dissection in 22 patients (19.1%). We compared these items between the learning set and the validation set. The median age at surgery was 67 years (IQR 60–72) in the validation set, significantly older than the learning set (*p* = 0.04). On the other hand, the proportion of those aged 75 years or older was 15.6% (n = 7) in the validation set, equivalent to the learning set (*p* = 0.47). There were no statistically significant differences in other patient backgrounds, tumor backgrounds, or surgical details.

**Table 1:** Comparison of clinical characteristics of the patients with rectal cancer between learning and validation set

Variable	Learning set (n = 115)	Validation set (n = 45)	*p* value
Patient characteristics			
Male sex, n (%)	73 (63.5)	29 (64.4)	0.909
Age at surgery, years; median [IQR]	62 [55–67]	67 [60–72]	0.044
≥ 75, n (%)	13 (11.3)	7 (15.6)	0.466
BMI (kg/m^2^), median [IQR]	22.5 [20.4–24.5]	22.5 [19.3–25.3]	0.974
≥ 25, n (%)	23 (20.0)	15 (33.3)	0.076
Neo-adjuvant therapy, n (%)	21 (18.3)	8 (17.8)	0.943
Chemoradiotherapy, n (%)	0 (0)	1 (2.2)	
Chemotherapy alone, n (%)	21 (18.3)	7 (15.6)	
Tumor characteristics			
Tumor distance from AV, cm; median [IQR]	10 [6–15]	10 [6–15]	0.425
T stage (0–2/3, 4)	60/55	21/24	0.532
N stage (0/1, 2)	74/41	27/18	0.609
M stage (0/1)	110/5	43/2	0.979
Surgical details			
Surgical approach (robot/laparoscopy)	24/91	15/30	0.099
Type of surgery (ISR/HAR, LAR)	13/102	1/44	0.068
Construction of diverting ileostomy, n (%)	48 (41.7)	14 (31.1)	0.216
Lateral lymph node dissection n (%)	22 (19.1)	6 (13.3)	0.387

Values are the number of patients unless indicated otherwise

*IQR* interquartile range, *BMI* body mass index, *AV* anal verge

### Outcomes of the LARS survey

The outcomes of the LARS survey are shown in Table 2. The median LARS score was not significantly different between the learning set and the validation set (the learning set, 27 [IQR 11–34]; the validation set, 25 [IQR 13–32]; *p* = 0.80). The incidence of major LARS was over 30% in both sets: 40.9% (n = 47) in the learning set and 33.3% (n = 15) in the validation set.

**Table 2:** Comparison of LARS score components for patients with rectal cancer between learning and validation set

Variable	Learning set (n = 115)	Validation set (n = 45)	*p* value
LARS score, median [IQR]	27 [11–34]	25 [13–32]	0.796
LARS categories, n (%)			
No LARS	45 (39.1)	15 (33.3)	
Minor LARS	23 (20.0)	15 (33.3)	
Major LARS	47 (40.9)	15 (33.3)	

*IQR* interquartile range, *LARS* low anterior resection syndrome

### The predictive model of major LARS

**Fig. 2:**
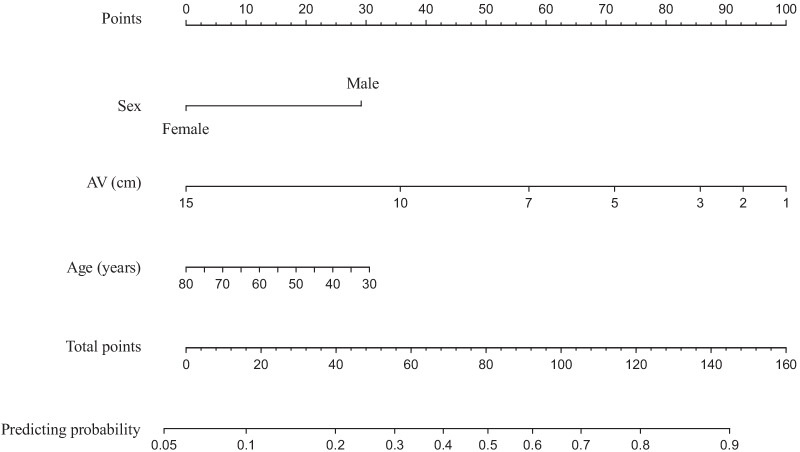
Nomogram for prediction of major LARS. *LARS* low anterior resection syndrome, *AV* anal verge

We performed a univariate and multivariate analysis using the learning set to identify and construct a nomogram of predictive factors for major LARS. A receiver operating characteristic analysis indicated the cut-off value of 8.5 cm for the tumor distance from the AV (area under the curve (AUC):0.77, *p* < 0.01; sensitivity: 0.73; specificity: 0.79; Additional file 1: Fig. S1). We performed a univariate analysis of the association of each clinical factor with the development of major LARS using the Mann–Whitney U test. We found that major LARS was significantly associated with sex, age, tumor distance from the AV, and type of surgery (Table 3). A multivariate analysis using logistic regression analysis was performed using the four factors with *p* values < 0.05 in the univariate analysis, and male sex (OR: 2.88, 95%CI 1.11–8.09, *p* = 0.03), age < 75 years (OR: 5.87, 95%CI 1.14–47.25, *p* = 0.03) and tumors located < 8.5 cm from the AV (OR: 7.20, 95% CI 2.86–19.49, *p* < 0.01) were independent predictors of major LARS (Table 4). We then constructed a nomogram of a predictive model for major LARS with the independent predictive variables, excluding ISR, which had *p* values > 0.05 in the multivariate analysis (Fig. 2). The related odds ratios used to create the nomogram were 2.70 for sex, 0.98 for age, and 0.78 for tumor location. The AUC of the nomogram for major LARS was 0.80 in the learning set, and the calibration was sound (Additional file 2: Fig. S2a). When we applied the nomogram to the validation set, the AUC was 0.76, and the calibration was also valid (Additional file 2: Fig. S2b).

**Table 3:** Univariate results for clinical characteristics associated with major LARS in the learning set

Variable	Category	No. of patients	Incidence of major LARS (%)	Univariate analysis^a^	
				OR (95% CI)	*p* value
Sex	Male	73	47.9	2.947 (1.297–7.127)	0.009
	Female	42	23.8	Reference	
Age, y	< 75	102	42.2	4.008 (1.010–26.765)	0.048
	≥ 75	13	15.4	Reference	
BMI, kg/m^2^	< 25.0	92	40.2	1.264 (0.495–3.411)	0.631
	≥ 25.0	23	34.8	Reference	
Tumor distance from AV, cm	< 8.5	48	67.4	9.375 (4.193–22.185)	< 0.001
	≥ 8.5	67	20.3	Reference	
Type of surgery	ISR	13	76.9	6.381 (1.818–29.840)	0.003
	HAR, LAR	102	34.3	Reference	

*OR* odds ratio, *CI* confidence interval, *LARS* low anterior resection syndrome, *BMI* body mass index, *AV* anal verge, *HAR* high anterior resection, *LAR* low anterior resection, *ISR* intersphincteric resection

^a^Mann-Whitney's U test

**Table 4:** Multivariate analysis of clinical characteristics associated with major LARS in the learning set

Factors	Multivariate analysis^a^	
	OR (95% CI)	*p* value
Male	2.883 (1.108–8.092)	0.030
Age < 75 years	5.871 (1.138–47.250)	0.033
Tumor distance from AV < 8.5 cm	7.201 (2.856–19.488)	< .001
Intersphincteric resection	2.518 (0.565–14.145)	0.233

*OR* odds ratio, *CI* confidence interval, *LARS* low anterior resection syndrome, *AV* anal verge

^a^Logistic regression analysis

## Discussion

Rectal cancer surgery is frequently associated with complications [15–17]. Defecation disorders are among the most frequent complications after rectal cancer surgery. The Japanese treatment guidelines recommend total mesorectal excision for patients with rectal cancer [18]. In addition, we are also actively working on lateral lymph node dissection and ISR to prevent local recurrences. However, these surgical treatments cause bowel dysfunction due to damage to the anal sphincter muscle and a reduction in rectal volume [19–21].

On the other hand, preoperative radiation therapy is recommended for patients with advanced rectal cancer in Western countries. It has been reported that this radiation therapy also leads to defecation disorders [13, 22]. The incidence of major LARS after rectal cancer surgery has been more than 40% [22–24], and the incidence of major LARS was similar in this study.

Defecation disorders impose limits on daily life activities and cause mental stress [25, 26]. Therefore, it is necessary to treat or prevent defecation disorders after rectal cancer surgery. One study reported that sacral nerve stimulation therapy could effectively alleviate defecation disorders [27]. However, there has been no evidence of an effective treatment for LARS. Recently, it has been reported that a nomogram may effectively prevent complications [28, 29]. In addition, the nomogram may be beneficial in counseling patients. Therefore, we expect that our nomogram can play an essential role in LARS therapy.

We identified three predictive factors for major LARS: male sex, age, and tumors location. Other reports have also placed these factors as predictors of defecation disorders. A feature of the nomogram is that the influence of each element can be visualized. As shown in Fig. 2, the distance from the anal margin to the tumor had the most potent effect on major LARS occurrences. This result is similar to the nomogram reported by Battersby et al. [13]. These results suggest that LARS severity might depend on the volume of the remaining rectum.

We found that age also strongly affected the development of major LARS in our nomogram. Older individuals have a weak anal sphincter muscle, which prevents gas or stool passage [30]. Therefore, fecal incontinence after rectal cancer surgery might occur more frequently in older patients. However, interestingly, we found that younger patients tended to have higher LARS scores in this study, consistent with findings in the previous European study [13]. There was no apparent difference in clinical characteristics when compared by age, as shown in Additional file 3: Table S1. On the other hand, comparing the results of the LARS survey, younger patients had higher scores than older patients in the question about the sense of urgency in defecation (Q5), although they showed little or no differences in the scores for questions 1–3, as shown in Additional file 3: Table S2. This result suggested that younger patients felt more fecal urgency than older patients, although rectal cancer surgery can cause stool and fecal incontinence, regardless of age.

Sex was also a correlating factor in the other report [13]. In the present study, men were more likely to develop major LARS. Generally, men have a narrower pelvic floor than women, making it more challenging to manipulate and putting more external stress on the surrounding tissues, including the pelvic floor muscles, which may have led to defecation problems. On the other hand, the nomogram reported by Battersby et al. suggests that women are more likely to develop major LARS and to have defecation disorders due to damage to the pelvic floor muscles caused by childbirth. Besides sex, several other factors caused our results to differ from Battersby et al. These may be due to differences in treatment strategies for rectal cancer between Japan and Europe and physical and lifestyle differences. The nomogram has the problem that results may differ depending on the subject's background, and the application of the nomogram needs to be carefully examined. The AUC of the European nomogram was 0.60 in the present study of 160 patients, and our nomogram showed higher accuracy than it.

A significant problem in this study is the sample size. The sample size used to create our nomogram was small compared with that reported by Battersby et al. The incidence of LARS was more than 50%, which, although feasible to analyze, may not have been sufficient to perform a detailed analysis. In particular, although we examined the impact on LARS of internal anal sphincter resection, which was not identified as a significant factor in the multivariate analysis, the sample size may have been inadequate given that only 13 patients underwent ISR. Other limitations are that we conducted this study at a single institution. The sample size used for validation may have been insufficient in terms of the versatility of the nomogram we created. We believe that a prospective, multicenter clinical study using our generated nomograms is needed to address these issues.

## Conclusions

We identified three independent predictors of major LARS for Japanese patients who undergo rectal cancer surgery: sex, the age at surgery, and the location of the tumor. We also constructed and validated a nomogram to predict major LARS from the above results. In the future, we will examine the applicability of the model developed in this study to other institutions and the impact of this model on rectal cancer treatment.

## Supplementary Information

**Additional file 1: Fig. S1**. ROC curves analysis to evaluate the predictive value of the tumor location for major LARS. ROC: receiver operating characteristic; LARS: low anterior resection syndrome; AV: anal verge; AUC: area under the curve.

**Additional file 1: Fig. S2**. Calibration curves for predicting major LARS in rectal cancer resection patients. Calibration curves for predicting major LARS in the learning set (a) and the validation set (b) are shown. The nomogrampredicted frequency of major LARS is plotted on the x-axis, and the actual observed frequency of LARS onset is plotted on the y-axis. LARS: low anterior resection syndrome.

**Additional file 3**. **Table S1:** Clinical characteristics of patients that underwent surgery for rectal cancer, classified by age. **Table S2:** Values of LARS questionnaire score components among patients after rectal cancer surgery, classified by age.

## Data Availability

The datasets used and analyzed during the current study are available from the corresponding author on reasonable request.

## Abbreviations

ISR: Intersphincteric resection
LARS: Low anterior resection syndrome
BMI: Body mass index
AV: Anal verge
IQR: Interquartile range
OR: Odds ratio
95% CI: 95% Confidence interval
AUC: Area under the curve
