# Supplementary figures and images for: Development and evaluation of a Japanese prediction model for low anterior resection syndrome after rectal cancer surgery
Source: BMC Gastroenterol. 2022 May 13;22:239. doi: 10.1186/s12876-022-02295-w (PMC9102936; doi:10.1186/s12876-022-02295-w)

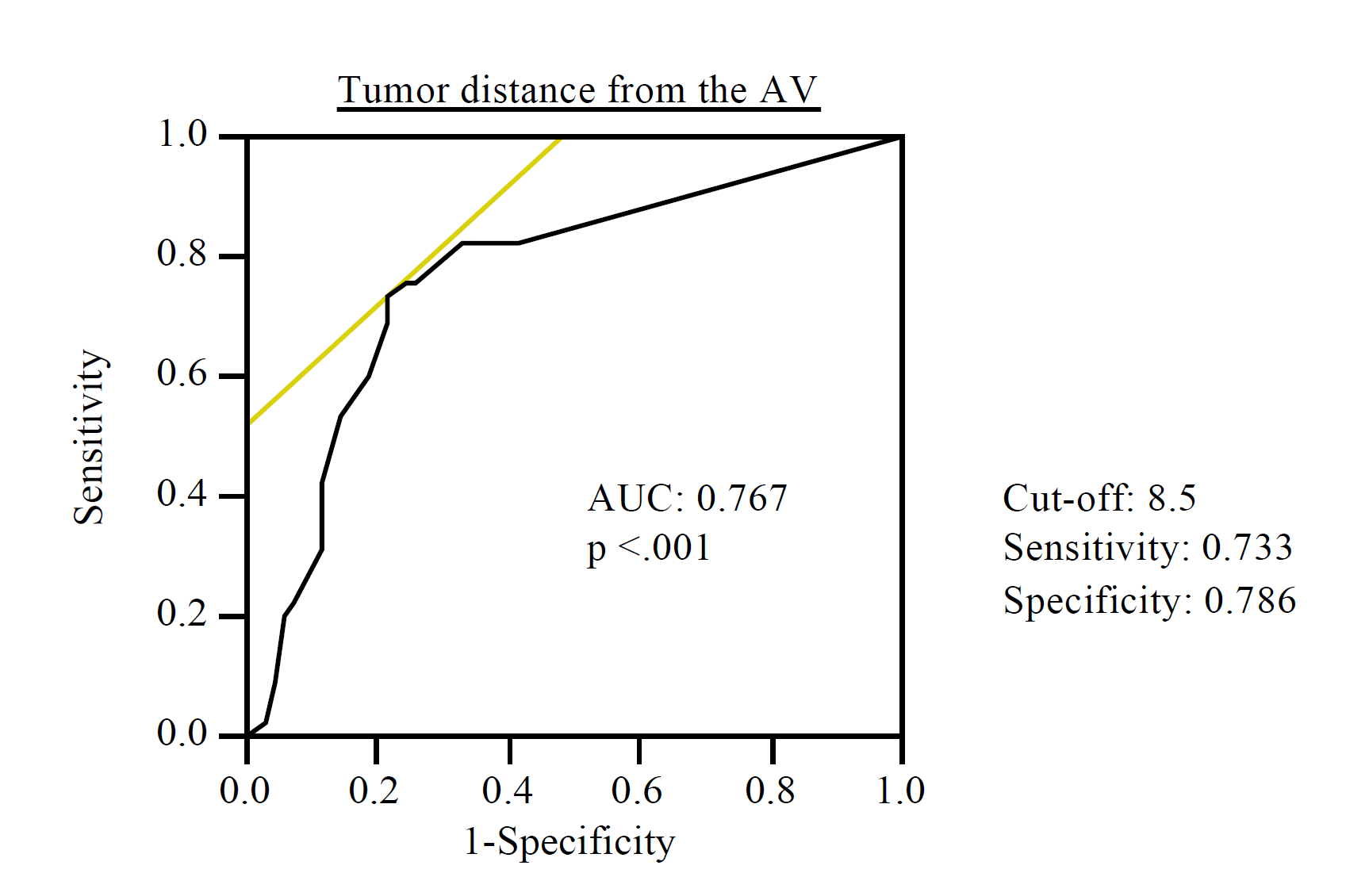

Supplement: Supplementary file 1 — Additional file 1: Fig. S1. ROC curves analysis to evaluate the predictive value of the tumor location for major LARS. ROC: receiver operating characteristic; LARS: low anterior resection syndrome; AV: anal verge; AUC: area under the curve. [file 12876_2022_2295_MOESM1_ESM.png]

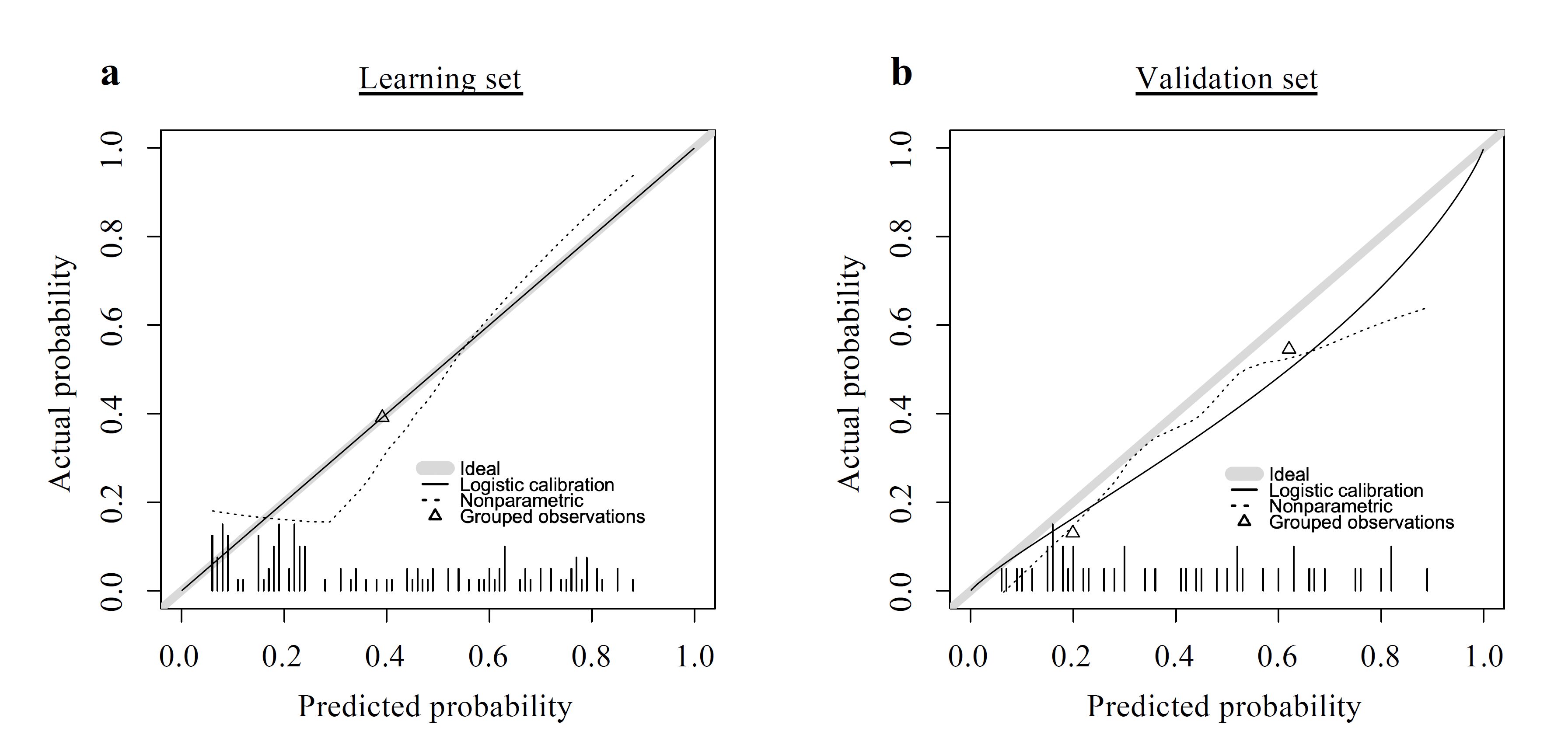

Supplement: Supplementary file 2 — Additional file 1: Fig. S2. Calibration curves for predicting major LARS in rectal cancer resection patients. Calibration curves for predicting major LARS in the learning set (a) and the validation set (b) are shown. The nomogrampredicted frequency of major LARS is plotted on the x-axis, and the actual observed frequency of LARS onset is plotted on the y-axis. LARS: low anterior resection syndrome. [file 12876_2022_2295_MOESM2_ESM.png]
