# Supplementary material for: Development and evaluation of a Japanese prediction model for low anterior resection syndrome after rectal cancer surgery
Source: BMC Gastroenterol. 2022 May 13;22:239. doi: 10.1186/s12876-022-02295-w (PMC9102936; doi:10.1186/s12876-022-02295-w)
Supplement: Supplementary file 3 — Additional file 3. Table S1: Clinical characteristics of patients that underwent surgery for rectal cancer, classified by age. Table S2: Values of LARS questionnaire score components among patients after rectal cancer surgery, classified by age. [file 12876_2022_2295_MOESM3_ESM.docx]

| **Additional file 3: Table S1** Clinical characteristics of patients that underwent surgery for rectal cancer, classified by age | | | |
| --- | --- | --- | --- |
| **Variable** | Age (years) | | |
|  | <75 (n = 140) | 75≤ (n = 20) | p value |
| Male sex, n (%) | 51 (36.4) | 7 (35.0) | 0.901 |
| BMI (kg/m^2^), median [IQR] | 22.2 [20.2-24.6] | 23.7 [21.7-25.3] | 0.216 |
| Neo-adjuvant therapy, n (%) | 25 (17.9) | 4 (20.0) | 0.817 |
| Tumor distance from AV, cm; median [IQR] | 10 [5.5-15] | 13.5 [6-15] | 0.381 |
| Laparoscopic surgery, n (%) | 106 (75.7) | 15 (75.0) | 0.945 |
| ISR, n (%) | 13 (9.3) | 1 (5.0) | 0.527 |
| Construction of diverting ileostomy, n (%) | 56 (40.0) | 6 (30.0) | 0.392 |
| Lateral lymph node dissection n (%) | 26 (18.6) | 2 (10.0) | 0.347 |
| Values are the number of patients, unless indicated otherwise. IQR: interquartile range; BMI: Body mass index; AV: Anal verge; ISR: intersphincteric resection | | | |

| **Additional file 3: Table S2** Values of LARS questionnaire score components among patients after rectal cancer surgery, classified by age | | | |
| --- | --- | --- | --- |
| Variable | Age (years) | | p value |
|  | <75 (n = 140) | 75≤ (n = 20) |  |
| Q1. Cannnot control flatus, average ± SD | 3.2 ± 3.2 | 2.6 ± 2.8 | 0.435 |
| Q2. Liquid stool leakage, average ± SD | 1.1 ± 1.4 | 0.6 ± 1.2 | 0.184 |
| Q3. Bowel movement frequency, average ± SD | 1.7 ± 1.7 | 2.1 ± 2.1 | 0.481 |
| Q4. Bowel movement within 1 h, average ± SD | 9.0 ± 3.6 | 6.0 ± 5.0 | 0.003 |
| Q5. Strong urge for bowel movement, average ± SD | 9.2 ± 6.7 | 4.6 ± 6.4 | 0.008 |
| Total score, , average ± SD | 24.1 ± 12.6 | 15.9 ± 12.5 | 0.010 |
| LARS: low anterior resection syndrome; SD: standard deviation | | | |
